# Comprehensive evaluation of diastolic function with MRI

**DOI:** 10.1186/1532-429X-11-S1-O95

**Published:** 2009-01-28

**Authors:** Thompson Richard, June Cheng Baron, Kelvin Chow, Jessica Scott, Ben Esch, Mark Haykowsky, Ian Paterson

**Affiliations:** 1 University of Alberta, Edmonton, AB Canada; 2 University of British Columbia, Vancouver, BC Canada

**Keywords:** Diastolic Dysfunction, Diastolic Function, Heart Failure Patient, Ventricular Relaxation, Diastolic Parameter

## Introduction

Diastolic dysfunction is a contributing factor in most cardiovascular diseases. For example, from the ischemic cascade, it is well known that ventricular relaxation is impaired prior to changes in systolic function. Diastolic parameters are predictive of outcome in acute MI [1], and a third to a half of all cases of heart failure have preserved LVEF (>50%) (HFpEF) [2]. The importance of diastolic dysfunction in the many manifestations of HFpEF is not well characterized. Clinical evaluation of diastolic function is predominantly by echocardiography, for which several conventional and novel quantitative measures of function are available (the vast majority of which are not routinely acquired using MRI). With its increasing use in clinical cardiology, and improvements in temporal resolution, it is now practical for MRI to provide an equivalent or superior assessment of diastolic function. We illustrate the measurement of conventional and novel diastolic parameters using universally available clinical pulse sequences.

## Methods

Diastolic parameters are measured in a controls (n = 10) and heart failure patients (n = 10) with diverse etiologies (ischemic and non-ischemic cardiomyopathies, 13% < EF < 67%). MRI studies consisted of conventional volumetric cines (SAX and LAX) for the measurement of ESV, EDV, SV (normalized to body surface area) and EF, phase contrast (basal SAX through-plane with V_enc_ = 120 cm/s and V_enc_ = 30–50 cm/s, 3 ch and 4 ch with in-plane velocities) and tissue tagging (5 SAX and LAX slices). Conventional diastolic parameters: E and A wave filling velocities (cm/s), mitral annular velocity (E' in cm/s), E/A ratio, E/E' ratio and inflow propagation velocity (V_p_ in cm/s). Additional parameters include the intraventricular (IVPG) and atrial (IAPG) pressure gradients (derived from in-plane blood velocities), peak torsion (deg) and rate of untwisting (deg/sec), peak diastolic radial velocity (ventricular average – cm/s), and peak diastolic circumferential strain rate (ventricular average, s^-1^). All tagged images were analyzed using a user-independent morphing approach. All studies were breath held with ECG gating (Siemens Sonata 1.5 T scanner).

## Results

**Figure 1:**
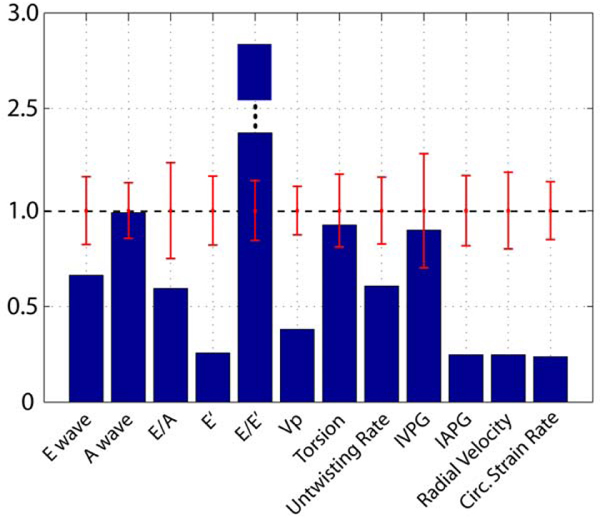
**Normalized diastolic parameters (heart failure patient)**.

Tables 1, 2 and 3 summarize the conventional volume and diastolic parameters (both conventional and novel measures) in the control and heart failure subjects. Figure 1 compares one failure case (ischemic cardiomyopathy with EF = 26%) with the control population using normalized diastolic parameters. The control population standard deviations for each parameter are shown, clearly illustrating that several diastolic parameters are abnormal, notably the conventional E' and E/E' values (most sensitive clinical measures of diastolic dysfunction [3]) and most of the novel measures in this subject. Similar striking patterns of abnormal diastolic function are seen in most heart failure patients in this study as indicated by Tables 2 and 3.

**Table 1:** Heart rate, volumes and function

	HR	EDVi (mL/m^2^)	ESVi (mL/m^2^)	Svi (mL/m^2^)	EF(%)
Control	67.1(14.0)	92.3(16.3)	35.7(9.0)	56.6(8.4)	61.6(3.7)
Patients	74.9(20.9)	126.4(88.5)	80.8(41.4)	45.6(21.7)	38.5(17.6)

**Table 2:** Conventional diastolic parameters

	E(cm/s)	A(cm/s)	E/A	E' (cm/s)	E/E'	Vp (cm/s)
Control	64.6(11.4)	34.6(5.0)	1.9(0.5)	14.4(2.6)	4.5(0.7)	57.8(7.3)
Patients	63.5(22.5)	40.8(13.4)	1.5(0.9)	9.7(6.5)	8.6(4.5)	32.5(13.7)

**Table 3:** Novel diastolic parameters

	IVPG_peak_ (mmHg)	IAPG_peak_ (mmHg)	Peak Torsion (deg)	Peak Untwisting Rate (deg/sec)	Radial Velocity (cm/s)	Circumferential Strain rate (s^-1^)
Control	2.9(0.9)	1.9(0.4)	11.1(2.1)	157.2(27.6)	4.4(0.9)	1.60(0.24)
Patients	3.3(2.3)	0.8(1.4)	8.4(4.3)	77.7(30.5)	2.3(1.0)	0.87(0.46)

## Conclusion

MRI can offer a comprehensive evaluation of diastolic function that is comparable or superior to echocardiography. In most heart failure patients the conventional and several novel measures could be measured using conventional pulse sequences, with arrhythmias being the most common technical limitation (2 of the 10 subjects were excluded due to arrhythmias). Using automated processing tools for tag and phase contrast data analysis, rapid and standardized processing is now feasible. In addition to superior LV volumes and function, MRI is the gold standard measure of LA volumes, which is sensitive to increased diastolic pressures, and delayed enhancement offers a measure of fibrosis, which is an important modulator of ventricular relaxation and stiffness and underlying cause of diastolic dysfunction.

## References

[1] Moller JE (2006). Circulation.

[2] Paulus WJ (2007). Eur Heart J.

[3] Kasner M (2007). Circulation.

